# Psychological Distress and Zika, Dengue and Chikungunya Symptoms Following the 2016 Earthquake in Bahía de Caráquez, Ecuador

**DOI:** 10.3390/ijerph14121516

**Published:** 2017-12-05

**Authors:** Anna M. Stewart-Ibarra, Anita Hargrave, Avriel Diaz, Aileen Kenneson, David Madden, Moory M. Romero, Juan Pablo Molina, David Macias Saltos

**Affiliations:** 1 Center for Global Health and Translational Science, SUNY Upstate Medical University, 750 East Adams St., Syracuse, NY 13210, USA; aileen.kenneson@yahoo.com (A.K.); romero.moory@gmail.com (M.M.R.); 2 Department of Medicine, University of California San Francisco, San Francisco, CA 94143, USA; anita.hargrave@ucsf.edu; 3 Department of Ecology, Evolution and Conservation Biology at Columbia University, New York, NY 10025, USA; avriel.diaz@gmail.com; 4 Walking Palms Global Initiative, Bahía de Caráquez 131401, Manabí Province, Ecuador; davidpatrickmadden@gmail.com; 5 Ministry of Health, San Vicente 131458, Manabí Province, Ecuador; molinajp2014@hotmail.com (J.P.M.); davidmacias126@gmail.com (D.M.S.)

**Keywords:** Zika virus, dengue, chikungunya, arbovirus, psychological distress, earthquake, Ecuador, natural disaster

## Abstract

On 16 April 2016, a 7.8 magnitude earthquake struck coastal Ecuador, resulting in significant mortality and morbidity, damages to infrastructure, and psychological trauma. This event coincided with the first outbreak of Zika virus (ZIKV) and co-circulation with dengue virus (DENV) and chikungunya virus (CHIKV). We tested whether the degree of psychological distress was associated with the presence of suspected DENV, CHIKV, ZIKV (DCZ) infections three months after the earthquake. In July 2016, 601 household members from four communities in Bahía de Caráquez, Manabí Province, Ecuador, were surveyed in a post-disaster health evaluation. Information was collected on demographics, physical damages and injuries, chronic diseases, self-reported psychological distress, and DCZ symptoms. We calculated the prevalence of arbovirus and distress symptoms by community. ANOVA was used to compare the mean number of psychological distress symptoms between people with versus without suspected DCZ infections by age, gender, community and the need to sleep outside of the home due to damages. The prevalence of suspected DCZ infections was 9.7% and the prevalence of psychological distress was 58.1%. The average number of psychological distress symptoms was significantly higher among people with suspected DCZ infections in the periurban community of Bella Vista, in women, in adults 40–64 years of age and in individuals not sleeping at home (*p* < 0.05). The results of this study highlight the need to investigate the interactions between psychological distress and arboviral infections following natural disasters.

## 1. Introduction

This study aims to describe the prevalence of psychological distress and arbovirus symptoms in vulnerable communities that were affected by the earthquake that occurred in Ecuador in April 2016. Since stress can affect the immune system adversely, we hypothesized that individuals who reported a greater degree of psychological distress would be more likely to report arbovirus symptoms.

**Figure 1:**
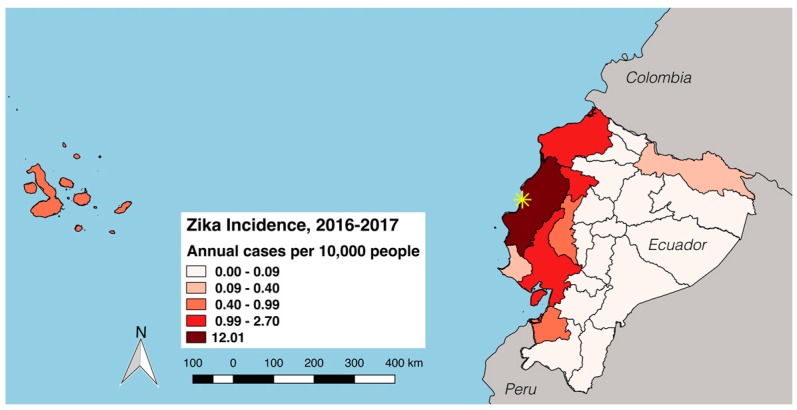
Annual ZIKV incidence per province in Ecuador, from epidemiological week (EW) 1 in 2016 to EW 38 in 2017 (cases per 10,000 people). The site of this study (Bahía de Caráquezf, Manabí Province) is indicated by the yellow asterisk. The earthquake epicenter was located 124 km northwest of Bahía de Caráquez. Data provided by the Ministry of Health of Ecuador [4].

**Figure 2:**
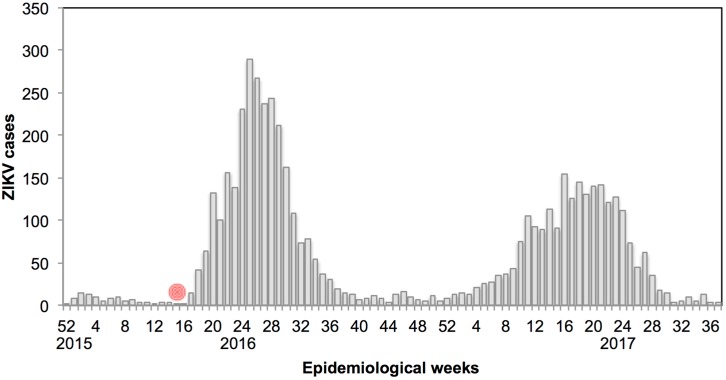
Weekly ZIKV cases in Ecuador through epidemiological week (EW) 38 in 2017. The date of the earthquake (EW 15, 2016) shown in red. In 2016, 85.1% of ZIKV cases depicted here (2510 of 2944 cases) were reported from Manabí Province, the location of this study. Data provided by the Ministry of Health of Ecuador [4].

*The earthquake and Zika epidemic in Ecuador.* On 16 April 2016, one of the most devastating earthquakes of the last century occurred in coastal Ecuador (magnitude 7.8 Richter scale). There were 660 reported fatalities, 4605 people with injuries, 30,223 people displaced, 9738 buildings damaged/destroyed and roughly 720,000 people requiring humanitarian assistance [1]. The earthquake triggered a major epidemic of Zika virus (ZIKV) [2], a major health concern due to the complications associated with infection, such as Guillain-Barre Syndrome and congenital syndrome [3]. Following the earthquake, the cumulative number of ZIKV cases in Ecuador increased from 103 cases in epidemiological week 14 of 2016 to 1275 cases in epidemiological week 25 of 2016, with 85% of all ZIKV cases in 2016 reported from Manabí Province, near the epicenter (Figure 1 and Figure 2) [4]. There were repeated aftershocks in affected areas for months after the initial event. These communities experienced unparalleled stress from the increased burden of disease coupled with the material, psychological and physical damage they endured.

ZIKV (family: *Flaviviridae*, genus: *flavivirus)* is transmitted to people by the *Aedes aegypti* and *Ae. albopictus* mosquitoes, the same vectors as the dengue virus (DENV 1–4, family: *Flaviviridae*, genus: flavivirus) and chikungunya virus (CHIKV, family: *Togaviridae*, genus: *alphavirus*). These arboviruses have a similar clinical presentation (e.g., acute febrile illness) and many infections are mild or subclinical [3,5,6]. Coastal Ecuador is historically hyper-endemic for DENV due to high densities of *Ae. aegypti* in urban areas [7,8,9,10]. *Ae. aegypti* is an anthropophilic mosquito vector, whose preferred larval habitat is standing water in man-made containers (e.g., 55 gallon drums, buckets, tires, flower vases) [5]. The risk of arboviral infections increased following the earthquake due, in part, to damages to the piped water system. As a result, people stored water in uncovered containers around the home, increasing the availability of mosquito larval habitat. Many people lost their homes and were forced to relocate to formal and informal tent camps, increasing their exposure to mosquito bites. Pre-existing poverty and other social vulnerabilities were exacerbated [11]. The earthquake also coincided with an exceptionally strong El Niño event, resulting in warmer than average air temperatures in coastal Ecuador, which increased the risk of arbovirus outbreaks [11,12,13,14].

In Ecuador, it is mandatory that DENV, CHIKV and ZIKV (DCZ) cases be reported to the Ministry of Health (MoH), which follows World Health Organization (WHO) guidelines for diagnosis and reporting. DCZ cases are diagnosed by clinical symptoms and epidemiological nexus; a subset of cases are diagnosed by laboratory assays at the National Public Health Research Institute (INSPI) of the MoH. Vector control and educational campaigns are the primary public health interventions to prevent and control disease outbreaks (e.g., chemical control and elimination of larval habitat, fogging and indoor residual spraying). In the period following the earthquake, the MoH conducted a campaign to distribute bed nets to pregnant women to prevent ZIKV transmission in the affected areas.

*Psychological distress and infectious diseases post-disasters.* Natural disasters bring with them damages to housing and infrastructure, lack of sanitation, crowding in formal and informal resettlements, and damage to health care facilities, especially in resource-limited settings. It is not surprising that increases in morbidity and mortality from infectious diseases have often been observed in their wake [15,16]. For example, in 2010, a major earthquake struck Haiti, taking the lives of an estimated 230,000 persons and displacing over 1.5 million others. The earthquake was followed by a devastating cholera (*Vibrio cholerae*) epidemic [17]. Similarly, after the 2004 floods in Bangladesh, there was a spike in enterotogenic *Escherichia coli* (ETEC) and *V. cholerae*, leading to an epidemic of diarrheal illness [18].

Populations affected by natural disasters may also experience psychological morbidity [19,20,21]. Earthquake survivors commonly suffer from post-traumatic stress disorder (PTSD) [20,22,23,24], characterized by re-experiencing the event, with persistent avoidance of factors associated with the event and negative thoughts/feelings after the trauma [25]. Anxiety, depression and suicidal ideation have also been noted to severely impact the lives of earthquake survivors [20,22]. Early studies in Colombia and Ecuador revealed the impact of natural disasters on mental health in Andean countries, with a higher prevalence of psychological distress in areas with more severe natural disasters [26,27,28]. In these populations, the most common psychological diagnoses post-disaster were PTSD and major depression.

Stress has been shown to significantly influence the response of the immune system by triggering the release of hormones and other substances in the brain. In addition, people’s behaviors often change in response to stressful situations, for example, changes in sleeping patterns, poor nutrition, and substance abuse [29]. Such changes can potentially alter the response of the immune system to infectious pathogens [30]. Stress has been associated with higher rates of respiratory tract infections, herpes virus flares and malignancies [30,31,32]. Early studies showed that psychological stress was associated in a dose-response relationship with increased risk of acute respiratory illness [33]. Higher cortisol levels, which can suppress the immune response, have been associated with more severe pneumonia and DENV infections [34,35]. Cellular immune dysfunction has also been noted among patients with PTSD [36]. Mouse model studies found that stress can increase morbidity due to viral infection [37,38,39].

Despite the robust literature supporting the surge in psychological distress and infectious diseases following certain natural disasters, there are very few studies that integrate these three areas. Here we present the results of an analysis of a cross-sectional survey on psychological distress and DCZ symptoms conducted in communities affected by the earthquake in Manabí Province, Ecuador, in July 2016, three months after the earthquake.

## 2. Methods

### 2.1. Study Area

Bahía de Caráquez (population 20,921) is a small tourist city located along the north-central coast of Ecuador in Manabí province (Figure 1). The city was heavily damaged in the earthquake. The public hospital was so severely damaged that it had to be closed. As a result, most health interventions in the communities affected by the earthquake were limited to primary care. ZIKV incidence rates in Manabí province remain the highest in the country (average annual incidence in 2016 and 2017: 12.0 cases per 10,000 people) [4].

### 2.2. Participants

Four communities in Bahía de Caráquez were included in this study: Jorge Lomas, Pajonal, La Merced and Bellavista. Jorge Lomas is an urban community located near the city center. Community members are a mix of local permanent residents and families with vacation homes. The majority of those surveyed were permanent residents, as most vacationers had fled the area. The primary income for local residents comes from a mix of urban jobs (teachers, shop owners, restaurant business, etc.). Pajonal is a rural fishing community. Nearby areas are being developed by private luxury housing companies along the coastline, providing jobs in construction. Some homes were damaged, but the community church, school and center remained intact. La Merced is a rural community that is located near a health center that was severely impacted by the earthquake. The community is located on the outskirts of Bahía de Caráquez, and many of the residents are employed by shrimp farms or are local fishermen. Following the earthquake many homes were damaged and water shortages resulted in increased water storage around homes. Bella Vista is a community located on a steep hillside at the urban periphery. For many years, this community has not been recognized as a formal urban settlement. Informal employment is the default source of income (e.g., fishing, construction, and petty trade work). We observed that most of the homes on the hillsides were completely demolished following the earthquake.

### 2.3. Data Source

De-identified data were provided by the Ministry of Health of Ecuador (Zone XI, San Vicente, Sucre, Manabí Province). Data were collected by Ministry of Health field technicians and disaster relief volunteers as part of a post-disaster health evaluation. Surveys were conducted from 19–25 July 2016. Homes were selected if household members were at home during the day when the technicians conducted face-to-face surveys. All household members were surveyed (N = 601), and the responsible adult in the home responded on behalf of young children. In most instances, the head of the household relayed information about people living in the household if they were not present. No identifiable human subject information was included in the dataset analyzed in this study. The local Ministry of Health approved of this study. The Institutional Review Board of SUNY Upstate Medical University (Syracuse, NY, USA) deemed that this study was exempt from review, as no identifiable information was included.

### 2.4. Measures

We analyzed the following individual-level data: demographics, basic medical history (pregnancy, chronic diseases, disabilities), the presence/absence of self-reported psychological distress symptoms post-earthquake (subjective feelings of stress, fear, insomnia, anorexia, chest pain, fear of being alone, anxiety, nightmares, headache, difficulty concentrating, hopelessness and sadness), damages to the home and physical injury due to the earthquake, whether individuals were sleeping at home or elsewhere, whether they had an episode of DCZ illness post-earthquake and the symptoms associated with the illness (headache, anorexia or nausea, conjunctivitis, rash, bleeding, vomiting, lethargy, abdominal pain, diarrhea, retro-orbital pain, muscle/joint pain), date of DCZ symptom onset, and whether they sought medical care. Psychological and DCZ symptoms were recorded if symptoms commenced after the April 2016 earthquake. Psychological distress symptoms were culturally adapted from the Anxiety and Depression Association of America. We classified an individual with a suspected DCZ infection if an individual reported fever plus two or more additional symptoms. This definition follows the WHO classification for dengue fever [5]. Damage to the home were defined as *mild* if occupants were able to return to the home immediately, *moderate* if some repairs had to be made before returning home, and *severe* if occupants had not yet been able to return.

### 2.5. Data Analysis

Statistical analyses were conducted using SAS 9.4 (SAS Institute Inc., Cary, NC, USA). We assessed whether the number of distress symptoms was associated with the presence/absence of suspected DCZ infections by study site, gender, age class, and whether the individual was sleeping at home. The variable “headache” was excluded from DCZ and distress symptoms due to a high degree of correlation. Bivariate analyses were conducted using *t*-tests (continuous variables) or Chi-squared tests (categorical variables), and comparisons across multiple groups were conducted by ANOVA. Adjustments were not made for multiple comparisons, and results should be interpreted accordingly.

## 3. Results

### 3.1. Demographics and Earthquake Damages

A total of 601 individuals from 160 households were included in this analysis, with 360 individuals (59.9%) from the two urban sites and 241 individuals (40.1%) from the two rural sites (Table 1). Participants were 28.9 years of age on average (SD = 21.2, median = 23); 48.4% were female and 51.6% were male. Eight women were pregnant, accounting for 5.4% of women of reproductive age (15 to 49 years of age). Five percent (30/601) of the population reported one or more disabilities (3 auditory, 7 visual, 5 mental, 12 physical, 3 not reported). One quarter (92/349, 26.4%) of the adult population (>18 years of age) reported one or more chronic health conditions (Table 1). Hypertension (50/349, 14.3%) and diabetes (32/349, 9.2%) were the most prevalent chronic health conditions in adults (see Supplementary Materials Table S1 for chronic conditions by site).

The majority of homes surveyed in this study suffered damages due to the earthquake (143/160, 89.4%) (Table 2). Almost half of the homes had severe damages (63/150, 42%) and as a result, individuals from almost half of the homes were sleeping somewhere other than at home (70/160, 43.8%). In one in five homes, someone was injured during the earthquake (30/160, 18.8%). Bella Vista suffered the greatest damages to homes (95.6% of homes damaged; 45.7% of damages were severe; 60.8% were not sleeping at home). Households in La Merced had the highest frequency of injuries (34% of homes with someone injured). Pajonal was the site with the least damage (61.9% of homes damaged; 52.6% of damages were mild/moderate; 9.5% were not sleeping at home).

### 3.2. Psychological Distress

More than half of the respondents had one or more symptoms of psychological distress (343/590, 58.1%, Table 3). The most frequently reported symptoms were fear (308/590, 52.2%), stress (98/590, 16.6%), insomnia (95/590, 16.1%) and monophobia (fear of being alone) (38/590, 6.4%). There were significant differences in the frequency of symptoms reported by study site (*p* < 0.05), with a higher mean number of distress symptoms in the rural sites (1.46 in La Merced and 1.31 in Pajonal, versus 0.85 in Bella Vista and 0.97 in Jorge Lomas). There was a significant difference in the number of distress symptoms by age class (*p* < 0.05). Adolescents (10–18 years) reported the greatest number of stress symptoms (mean = 1.46, SD = 1.75) and young adults (19–39 years) the least (mean = 0.84, SD = 1.18). There were no significant differences in the number of stress symptoms by gender (female: mean = 1.20, SD = 1.33; male: mean = 0.98, SD = 1.38, *p* > 0.05) or by sleeping at home versus not at home (at home: mean = 1.18, SD = 1.41; not at home: mean = 1.03, SD = 1.35, *p* > 0.05). We calculated the average number of psychological distress symptoms for adolescents sleeping at home versus not sleeping at home and found that there were no significant differences (at home: mean = 1.51, SD = 1.94; not at home: mean = 0.97, SD = 1.16, *p* = 0.07).

### 3.3. Arbovirus Symptoms

We identified 50 individuals with a suspected DCZ infection post-earthquake (50/601, 9.7%) (Table 4). No pregnant women had a suspected DCZ infection post-earthquake. Of those with suspected DCZ infections, 62% had sought medical care. The most frequently reported symptoms were fever (66/601, 11.0%), headache (65/601, 10.8%), muscle/joint pain (61/601, 10.1%), and rash (47/601, 7.8%). There was a significant difference in the prevalence of suspected DCZ infections among sites (*p* < 0.05). The highest prevalence of suspected DCZ infections was found in Bella Vista (28/274, 10.2%), followed by La Merced (12/171, 7%). Individuals who reported that they were not sleeping at home were significantly more likely to have suspected DCZ infections (90/269, 33%) than those who were sleeping at home (47/326, 14.4%) (*p* < 0.00001). There were no significant differences in the frequency of suspected DCZ infections by age group or by gender.

### 3.4. Distress and Arboviral Symptoms 

**Figure 3:**
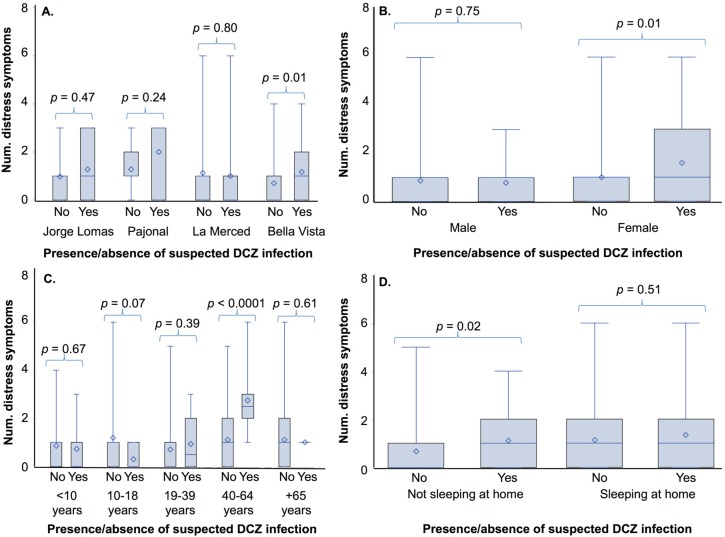
Average number of psychological distress symptoms among participants with and without suspected dengue, chikungunya, or Zika (DCZ) infections by (**A**) study sites, (**B**) gender, (**C**), age classes and (**D**) and whether or not the individual was sleeping at home or elsewhere. Significant differences indicated by *p* < 0.05.

We evaluated whether the average number of psychological distress symptoms varied in individuals with and without suspected DCZ infection by study site, gender, age and sleeping at home (Table 5, Figure 3). In the comparison across study sites, we found that in the community of Bella Vista, individuals with suspected DCZ infections reported a greater mean number of psychological distress symptoms than those without suspected DCZ infections (with DCZ: mean = 1.18, SD = 1.18; without DCZ: mean = 0.70, SD = 0.92, *p* = 0.01). Although individuals with suspected DCZ infection in Jorge Lomas and Pajonal also reported a greater average number of distress symptoms, the findings did not reach statistical significant (*p* > 0.05). In a comparison by gender, we identified a significantly greater number of distress symptoms in women with suspected DCZ infections (with DCZ: mean = 1.60, SD = 1.55; without DCZ: mean = 1.00, SD = 1.09, *p* = 0.01). There were no significant differences in men (*p* > 0.05). In a comparison across age classes, individuals 40–64 years of age with suspected DCZ infections had a significantly greater average number of distress symptoms (with DCZ: mean = 2.75, SD = 1.29; without DCZ: mean = 1.12, SD = 1.17, *p* < 0.0001). The differences were not significant in the other age classes (*p* > 0.05). Individuals with suspected DCZ infections who were not sleeping at home had significantly greater number of distress symptoms (with DCZ: mean = 1.10, SD = 1.18; without DCZ: mean = 0.66, SD = 0.91, *p* = 0.02). There were no significant differences in individuals sleeping at home (*p* > 0.05).

## 4. Discussion

This investigation of recent earthquake survivors sheds light on the degree of psychological distress and its association with suspected arbovirus infections following a natural disaster. This study is important given the unique nature of the co-occurrence of a severe earthquake event and the ZIKV epidemic. The earthquake occurred three and a half months after the first ZIKV cases were reported in Ecuador and triggered a major outbreak of ZIKV, in addition to outbreaks of DENV and CHIKV. In 2016, a total of 14,150 DENV cases [40], 2025 CHIKV cases [41] and 3531 ZIKV cases were reported in Ecuador [42]. We report positive associations between the degree of psychological distress and the presence of suspected dengue, chikungunya, Zika (DCZ) infections among affected populations near the earthquake epicenter. This underscores the need for an integrated approach to disaster relief interventions, which includes mental health professionals as well as health professionals with training in diagnosing and treating infectious diseases in post-disaster settings.

### 4.1. Psychological Distress

We found a high prevalence of psychological distress (58.1%) three months following the earthquake. This is consistent with prior studies that documented the effects of earthquakes and other natural disasters on mental health in Andean and other resource-limited countries. In a study conducted in Colombia seven to ten months after a volcanic eruption, approximately half of the individuals residing in resettlement camps and individuals visiting outpatient clinics reported symptoms of emotional distress [26]. In northern Ecuador, three months after an earthquake in 1987, 38% of adults who visited outpatient clinics had signs of psychological distress. This population was much less affected than the population surveyed here, as there was no loss of life in the study area. Investigations of earthquake survivors in Peru (five months post event), found that 25.2% of adults had PTSD [43]. In China, the prevalence of PTSD post-earthquake ranged from 44 to 60.4% [44], and the prevalence of psychological distress symptoms in adults following an earthquake in India was 59% [45]. The most common psychological diagnoses for earthquake survivors were PTSD and major depression [24,28,45]. Most studies suggest that PTSD symptoms decrease within the first two years following the event [23,46,47,48,49]. The risk of psychological distress has been shown to increase with proximity to the epicenter, by gender (female), in individuals who lack of social support, in individuals who lost their homes, those who suffered injuries and those who lacked access to food and water, among other factors [21,26,43,44,45].

In this study individuals from rural communities and adolescents reported a greater number of psychological distress symptoms. Studies have shown that PTSD symptoms are common in adolescents who have survived earthquakes, with prevalence rates ranging from 16.9 to 70.3% [23,50,51,52]. We observed that in people in rural communities felt isolated from earthquake relief efforts, which were focused in the more populated urban areas. It is possible that the distress that we documented may reflect pre-existing levels of distress in rural areas. A prior study on mental health in rural coastal Ecuador found that older adults (>59 years) had a 12% prevalence of depression, 15% prevalence of anxiety, and 5% prevalence of stress [53]. In the same age group, individuals in this study self-reported a 24.6% prevalence of stress, 4.9% prevalence of anxiety and 4.9% prevalence of hopelessness/sadness. Fear, the most common symptom documented here, was not reported as a separate entity in the prior study. These results suggest that adolescents and rural communities should be considered as part of targeted mental health interventions in a post-disaster setting.

### 4.2. Arbovirus Symptoms

We found that one in ten individuals (9.7%) met our definition for a suspected DCZ infection in the three months following the earthquake. These prevalence estimates are comparable to DENV prevalence estimates from an active surveillance study in southern coastal Ecuador [54], which reported a 8.9% prevalence of acute symptomatic (apparent) DENV infections in homes near confirmed DENV infections. The prevalence of suspected DCZ infections was greatest in the community of Bella Vista (10.2%) and in individuals who reported that they were not sleeping at home (33.0%). Periurban communities are often at greatest risk of acquiring *Ae. aegypti* transmitted diseases due to ample mosquito larval habitat near homes (e.g., discarded containers, tires, water storage drums), high population density, and substandard housing conditions which permit entry of the mosquito vector into the home [5,55,56,57]. People not sleeping at home were likely at greater risk of exposure to infectious mosquito bites, since in many instances, they were sleeping outdoors in crowded, informal tent camps.

Differential diagnosis of DCZ infections in a post-disaster setting is a major challenge, since these diseases are transmitted by the same mosquito vector, they co-occur in the same geographic region, the clinical presentation is similar, and there may be limited/no access to diagnostic tests. This study was limited by lack of laboratory confirmation of DCZ infections. For this reason, we do not differentiate between the infections; we report of the prevalence of DCZ symptoms and the prevalence of suspected DCZ infections. In an attempt to take a more rigorous approach to the clinical diagnosis, we applied the WHO guidelines for the diagnosis of DENV (fever plus two or more other listed symptoms) to define suspected DCZ infections. The guidelines for CHIKV and ZIKV diagnosis are less strictly defined; therefore, this study likely underestimated the prevalence of viral illness. It is also possible that we captured other infections that cause similar symptoms, such as respiratory infections, typhus fever, leptospirosis, or malaria [58]. In 2016, in Manabí Province, the following cases were reported: 5775 cases of DENV, 2509 cases of ZIKV, 339 cases of CHIKV, 21 cases of leptospirosis, 142 cases of typhoid and paratyphoid fever, 2279 cases of pneumonia, and 5 cases of malaria [59,60]. This suggests that DCZ infections were among the most common causes of febrile illness, although other infectious pathogens were also reported.

We found that a surprisingly high proportion of individuals with suspected DCZ infections had sought medical care (62.0%). This is much higher than has been observed in prior studies in Ecuador, which found that DENV infections are greatly underreported due to subclinical or mild clinical presentation of the disease and low health care seeking behavior [54,61,62]. The high proportion of individuals who had sought health care likely reflects effective coverage of primary medical care by the MoH and other disaster relief groups post-earthquake. 

### 4.3. Distress and Arbovirus Symptoms

We found that certain subgroups were more likely to have suspected DCZ infections if they reported a greater number of psychological distress symptoms, including residents of the periurban community of Bella Vista, women, individuals 40 to 64 years of age, and those who reported not sleeping at home. Bella Vista, with its precarious geography and weak housing construction, experienced the greatest damages to homes. Bella Vista also had the highest prevalence of DCZ symptoms, consistent with the urban habitat preferences of the *Ae. aegypti* mosquito. These findings suggest that periurban communities exposed to natural disasters face a double burden of psychological distress and arbovirus infections. The effects of stress may have increased people’s susceptibility to DCZ infections due to changes in behaviors that lowered their immune response. In a periurban setting, we observed that individuals were exposed to a host of environmental stressors, such as loud noises, which could disrupt their sleeping habits.

Adults (40–64 years) with a greater number of psychological distress symptoms were more likely to have suspected DCZ infections. Adults are generally the heads of their households and thus, were under greater stress to care for themselves and their family during and after the earthquake. During the 2015 CHIKV epidemic, older adults were the group most likely to have acute symptomatic CHIKV infections [54]. This may also have been true for ZIKV, as this was a new infection passing through a completely susceptible population.

Women with a greater number of distress symptoms were also more likely to have suspected DCZ infections. Prior studies suggest that women are at greater risk of psychological distress post-disaster [21]. The average number of distress symptoms in women (mean = 1.2) was greater than in men (mean = 0.98), although the differences were not statistically significant. In Ecuador, women are responsible for the health and wellbeing of their families, and prior studies found that stress and depression in Ecuadorian women was associated with excessive social responsibilities [63]. We observed that women leaders were exceptionally stressed in the post-disaster period, due to the demands to care for their families and community, which may have resulted in a lack of self-care. Women may have also had additional fear and/or anxiety around pregnancy and congenital malformations associated with the emerging epidemic of ZIKV. In Ecuador, family planning measures can be difficult to access [64] and elective abortions are criminalized [65]. The burden placed on women who lack adequate access to family planning in ZIKV endemic areas as been documented in similar Latin American countries [66,67].

The association between increased psychological distress and suspected DCZ infections among those who reported not sleeping at home is likely multifactorial. People did not sleep at home if their house was severely damaged and for fear of aftershocks. In the days following the earthquake, community members reported sleeping on the streets wherever they could find space. Afterwards, people constructed makeshift tents or moved into formal encampments set up by relief agencies. These experiences likely increased their exposure to the mosquito vector and resulted in poorer self-care, and psychological distress.

Stress can modulate the immune system through direct and indirect mechanisms [29]. The direct effects of stress on the immune system occur when the brain releases hormones and other substances that affect the immune response. Indirect effects occur when people’s behaviors change in response to stressful situations (e.g., changes in sleeping patterns, poor nutrition, substance abuse). The effects of stress on the immune system depends on the nature of the stressor (e.g., acute short-term stress versus chronic stress) and the type of adverse event. For example, loss and bereavement can increase cortisol levels, whereas PTSD can decrease cortisol levels [29]. We postulate that communities affected by the Ecuadorian earthquake simultaneously experienced ongoing loss and PTSD. Additional studies are needed to understand the effects of stress on the immune system and on susceptibility to infectious diseases in post-disaster settings. At a minimum, we can say that certain sub-groups appear to be more susceptible to the double burden of psychological distress and arbovirus infection. 

### 4.4. Limitations

This study represents the results of a rapid post-disaster health assessment conducted at one time point in four communities affected by the 2016 earthquake in Ecuador. As study participants were asked about their psychological distress and DCZ symptoms during the three-month period post-earthquake, it is possible that there were memory biases. Galea et al. (2005) present the epidemiological challenges of interpreting data on psychological distress collected after disasters [21]. They indicate that in an ideal setting, PTSD would be diagnosed by a mental health professional using the Diagnostic and Statistical Manual of Mental Disorders (DSM) criteria and instruments. However, in many real-world post-disaster settings, public health technicians conduct rapid health evaluations. This evaluation did include many of the key factors in the diagnosis of depression, anxiety and PTSD and was implemented by MoH technicians to be culturally sensitive and appropriate. However, given the nature of the survey, we were not able to distinguish between varying degrees of psychological distress (e.g., more or less fear or anxiety). For this reason, we report the presence/absence of psychological distress symptoms and the number of symptoms, rather than a definitive psychological diagnosis.

Since the survey was conducted at one time point post-earthquake, we report the point prevalence of psychological distress symptoms and suspected DCZ infections, rather than incidence [21]. A cohort study with data pre-disaster would have provided data on incidence. Surveys could have been conducted in communities with similar social profiles that were not affected by the earthquake; however, it would have been difficult to find such communities, because the 2016 earthquake affected such a large geographic area. Our study was also unique in that it occurred during the time of the first ZIKV epidemic in Ecuador.

Because our research was observational, we report a positive association between the degree of psychological distress and the presence of suspected DCZ infections but we cannot assign causality. Increased stress could increase the likelihood of a viral infection and a viral infection could lead to increased stress, resulting in a positive feedback loop. Longitudinal cohort studies are needed to address this question. Cumulative, long-term stress could be measured via hair cortisol [68,69,70] combined with validated psychosocial assessment tools to provide much more robust indicators of psychological distress following natural disasters or other stressful events.

## 5. Conclusions

The 2016 earthquake in Ecuador triggered an outbreak of ZIKV in coastal communities. In this study, we assessed whether individuals with high levels of psychological distress reported more symptoms compatible with ZIKV and other arboviral infections three months after the earthquake. The results of this study indicate a positive association between the degree of psychological distress and the presence of suspected arboviral (dengue, chikungunya, Zika) infections in a periurban community, women, adults (40–64 years) and individuals who, three months post-disaster, were still not sleeping at home. Future studies are needed to investigate how psychological distress from catastrophic events influences people’s capacity to mount an effective immune response to infectious pathogens, and how infectious diseases affect psychological distress. These findings highlight the need for integrated health responses following natural disasters that include healthcare professionals skilled in assessing and managing infectious diseases and psychological distress.

## Figures and Tables

**Table 1:** Demographic and clinical characteristics of study participants by site.

Variables	Bella Vista	Jorge Lomas	La Merced	Pajonal	Total
Setting	Urban	Urban	Rural	Rural	-
All participants (N)	274	86	171	70	601
Adults (>18 years) (% of N)	149 (54.4%)	62 (72.1%)	99 (57.9%)	39 (55.7%)	349 (58.1%)
Women (15 to 49 years) of reproductive age (% of N)	70 (25.5%)	24 (27.9%)	34 (19.9%)	21 (30.0%)	149 (24.8%)
Age, mean (SD), years	34.1 (22.6)	27.6 (20.8)	31.6 (22.5)	25.9 (19.5)	28.9 (21.2)
Female sex (% of N)	132 (48.2%)	48 (55.8%)	78/167 (46.7%)	31 (44.3%)	289/597 (48.4%)
Pregnant (% of women of reproductive age)	3 (4.30%)	1 (4.2%)	3 (8.8%)	1 (4.8%)	8 (5.4%)
Disabled (% of N)	12 (4.4%)	4 (4.7%)	12 (7.0%)	2 (2.9%)	30 (5.0%)
Hypertension (% of adults)	19 (12.8%)	8 (12.9%)	18 (18.2%)	5 (12.8%)	50 (14.3%)
Diabetes (% of adults)	13 (8.7%)	3 (4.8%)	14 (14.1%)	2 (5.1%)	32 (9.2%)
Any chronic disease (% of adults)	41 (27.5%)	9 (14.5%)	34 (34.3%)	8 (20.5%)	92 (26.4%)

**Table 2:** Earthquake damages. Households (n and %) that reported that someone was injured, whether household members were sleeping at home, and damages to the home as a result of the earthquake.

Damages	Bella Vista	Jorge Lomas	La Merced	Pajonal	Total
	N = 74	N = 21	N = 44	N = 21	N = 160
Someone in the home was physically injured.	10/74 (13.5%)	4/21 (19.0%)	15/44 (34.0%)	1/21 (4.8%)	30/160 (18.8%)
Not sleeping at home due to damages.	45/74 (60.8%)	4/21 (19.0%)	19/44 (43.2%)	2/21 (9.5%)	70/160 (43.8%)
Home was damaged.	71/74 (95.9%)	19/21 (90.5%)	40/44 (90.9%)	13/21 (61.9%)	143/160 (89.4%)
Mild damage *	19/70 (27.1%)	13/19 (68.4%)	15/42 (35.7%)	8/19 (42.1%)	55/150 (36.7%)
Moderate damage *	19/70 (27.1%)	3/19 (15.8%)	8/42 (19.0%)	2/19 (10.5%)	32/150 (21.3%)
Severe damage *	32/70 (45.7%)	3/19 (15.8%)	19/42 (45.2%)	9/19 (47.4%)	63/150 (42.0%)

* The proportion of respondents with mild, moderate and severe damages to their homes were calculated based on the number of respondents that had homes that were damaged and classified (Bella Vista N = 70, Jorge Lomas N = 19, La Merced N = 42, Pajonal N = 19). Damage to the home were defined as *mild* if occupants were able to return to the home immediately, *moderate* if some repairs had to be made before returning home, and *severe* if occupants had not yet been able to return.

**Table 3:** Prevalence of psychological distress symptoms by study site. Respondents (n and %) who reported distress symptoms by site.

Symptoms	Bella Vista	Jorge Lomas	La Merced	Pajonal	Total
	N = 263	N = 86	N = 171	N = 70	N = 590
Fear	114 (43.3%)	49 (57.0%)	89 (52.0%)	56 (80.0%)	308 (52.2%)
Stress	42 (16.0%)	13 (15.1%)	25 (14.6%)	18 (25.7%)	98 (16.6%)
Insomnia	29 (11.0%)	19 (22.1%)	38 (22.2%)	9 (12.9%)	95 (16.1%)
Monophobia (fear of being alone)	11 (4.2%)	0	21 (12.3%)	6 (8.6%)	38 (6.4%)
Anorexia	8 (3.0%)	0	12 (7.0%)	3 (4.3%)	23 (3.9%)
Headache	4 (1.5%)	0	17 (9.9%)	0	21 (3.6%)
Anxiety	12 (4.6%)	2 (2.3%)	6 (3.5%)	0	20 (3.4%)
Angina (chest pain)	0	0	12 (7.0%)	0	12 (2.0%)
Hopelessness	1 (0.4%)	0	10 (5.8%)	0	11 (1.9%)
Sadness	2 (0.8%)	0	8 (4.7%)	0	10 (1.7%)
Difficulty concentrating	1 (0.4%)	0	8 (4.7%)	0	9 (1.5%)
Nightmares	3 (1.1%)	0	3 (1.8%)	0	6 (1.0%)
1 or more symptom	138 (52.5%)	50 (58.1%)	99 (57.9%)	56 (80.0%)	343 (58.1%)
Mean number of symptoms (SD)	0.85 (1.73%)	0.97 (1.04%)	1.46 (1.87%)	1.31 (1.03%)	1.07 (1.29%)

**Table 4:** Prevalence of dengue, chikungunya or Zika fever (DCZ) symptoms by study site; respondents (n and %) who reported DCZ symptoms in the post-earthquake period, those who were defined as suspected DCZ infections (fever plus two additional symptoms), and individuals with suspected DCZ infections who sought medical care.

Symptoms	Bella Vista	Jorge Lomas	La Merced	Pajonal	Total
	N = 274	N = 86	N = 171	N = 70	N = 601
Fever	37 (13.5%)	9 (10.5%)	16 (9.4%)	4 (5.7%)	66 (11.0%)
Headache	35 (12.8%)	7 (8.1%)	20 (11.7%)	3 (4.3%)	65 (10.8%)
Muscle/joint pain	37 (13.5%)	9 (10.5%)	16 (9.4%)	4 (5.7%)	61 (10.1%)
Rash	26 (9.5%)	4 (4.7%)	17 (9.9%)	0	47 (7.8%)
Anorexia or nausea	9 (3.3%)	1 (1.2%)	4 (2.3%)	0	14 (2.3%)
Abdominal pain	8 (2.9%)	1 (1.2%)	4 (2.3%)	0	13 (2.2%)
Vomiting	8 (2.9%)	1 (1.2%)	3 (1.8%)	0	12 (2.0%)
Conjunctivitis	7 (2.6%)	1 (1.2%)	3 (1.8%)	0	11 (1.8%)
Retro-orbital pain	5 (1.8%)	1 (1.2%)	4 (2.3%)	0	10 (1.7%)
Diarrhea	4 (1.5%)	1 (1.2%)	4 (2.3%)	0	9 (1.5%)
Lethargy	3 (1.1%)	0	0	0	3 (0.5%)
Bleeding	1 (0.4%)	0	0	0	1 (0.2%)
Any symptom	49 (17.9%)	9 (10.5%)	26 (15.2%)	4 (5.7)	88 (14.6%)
Suspected DCZ infection (fever +2 symptoms)	28 (10.2%)	7 (0.8%)	12 (7.0%)	3 (4.3)	50 (9.7%)
Mean num. symptoms (SD)	3.6 (2.1%)	3.7 (2.3%)	3.4 (2.6%)	2.5 (1.0%)	3.4 (2.2%)
Those with suspected DCZ infections who sought medical care	17/28 (60.7%)	4/7 (57.1%)	10/12 (83.3%)	0	31/50 (62.0%)

**Table 5:** Distress symptoms reported by participants with versus without suspected dengue, chikungunya, Zika fever (DCZ) infections following the earthquake. Means and standard deviations (SD) shown. Significant differences indicated by *p* < 0.05.

**A. Sites**	**All**	**Bella Vista**	**Jorge Lomas**	**La Merced**	**Pajonal**
	**N = 464**	**N = 203**	**N = 72**	**N = 122**	**N = 67**
DCZ (SD)	0.95 (1.19)	0.70 (0.92)	0.99 (1.03)	1.12 (1.60)	1.28 (1.00)
No DCZ (SD)	1.20 (1.34)	1.18 (1.18)	1.29 (1.29)	1.00 (1.81)	2.00 (1.73)
*p*-value	*p* = 0.16	*p* = 0.01	*p* = 0.58	*p* = 0.80	*p* = 0.24
**B. Gender**	**Male**	**Female**			
	**N = 268**	**N = 245**			
DCZ (SD)	0.88 (1.24)	1.00 (1.09)			
No DCZ (SD)	0.80 (0.96)	1.60 (1.55)			
*p*-value	*p* = 0.75	*p* = 0.01			
**C. Age classes**	**<10 years**	**10–18 years**	**19–39 years**	**40–64 years**	**65+**
	**N = 130**	**N = 122**	**N = 178**	**N = 110**	**N = 61**
DCZ (SD)	0.86 (0.97)	1.19 (1.53)	0.69 (0.94)	1.12 (1.17)	1.10 (1.36)
No DCZ (SD)	0.73 (1.01)	0.30 (0.48)	0.93 (1.14)	2.75 (1.29)	1.00 (0)
*p*-value	*p* = 0.67	*p* = 0.07	*p* = 0.39	*p* < 0.0001	*p* = 0.61
**D. Sleeping at home**	**Not sleeping at home**	**Sleeping at home**			
	**N = 297**	**N = 201**			
DCZ (SD)	0.66 (0.91)	1.14 (1.30)			
No DCZ (SD)	1.10 (1.18)	1.33 (1.56)			
*p*-value	*p* = 0.02	*p* = 0.51			

## Supplementary Materials

The following are available online at www.mdpi.com/1660-4601/14/12/1516/s1, Table S1: The frequency and prevalence of chronic health conditions reported by study site.
