# Supplementary material for: Psychological Distress and Zika, Dengue and Chikungunya Symptoms Following the 2016 Earthquake in Bahía de Caráquez, Ecuador
Source: Int J Environ Res Public Health. 2017 Dec 5;14(12):1516. doi: 10.3390/ijerph14121516 (PMC5750934; doi:10.3390/ijerph14121516)
Supplement: Supplementary file 1 [file ijerph-14-01516-s001.pdf]

Supplementary Table S1. The frequency and prevalence of chronic health conditions reported by study site.

Chronic Condition	Jorge Lomas<br>N = 86	Pajonal<br>N = 70	La Merced<br>N = 171	Bella Vista<br>N = 274	Total<br>N = 601	P-value
Hypertension	8 (9.3%)	5 (7.1%)	18 (10.5%)	21/273 (7.59%)	52/600 (8.67%)	0.724
Diabetes	3 (3.5%)	2 (2.9%)	15 (8.8%)	13 (4.7%)	33 (5.5%)	0.142
Heart attack	0 (0%)	0 (0%)	4 (2.3%)	9 (3.3%)	13 (2.2%)	0.164
Respiratory illness	0 (0%)	0 (0%)	0 (0%)	1 (0.4%)	1 (0.2%)	0.754
Cancer, any	0 (0%)	0 (0%)	0 (0%)	0 (0%)	0 (0%)	N/A
Brain tumor	0 (0%)	0 (0%)	3 (1.8%)	0 (0%)	3 (0.5%)	0.056
Serious brain injury	0 (0%)	0 (0%)	0 (0%)	1 (0.4%)	1 (0.2%)	0.754
Malformation of brain venous artery	0 (0%)	0 (0%)	0 (0%)	1 (0.4%)	1 (0.2%)	0.754
Aneurism of thorax or abdomen	0 (0%)	3 (4.3%)	5 (2.9%)	17 (6.2%)	25 (4.2%)	0.064
Other chronic illness	4 (4.7%)	6 (8.6%)	2(1.2%)	11/270 (4.1)	23/597 (3.9%)	0.052
Any chronic condition	13 (15.2%)	13 (18.6%)	36 (21.0%)	54/270 (20.0%)	116/601 (19.3%)	0.716
Number of chronic conditions	Range = 0–2<br>Median = 0<br>Mean = 0.17<br>SD = 2.56	Range = 0–3<br>Median = 0<br>Mean = 0.23<br>SD = 2.91	Range = 0–4<br>Median = 0<br>Mean = 0.27<br>SD = 2.70	Range = 0–4<br>Median = 0<br>Mean = 0.27<br>SD + 0.61	Range = 0–4<br>Median = 0<br>Mean = 0.25<br>SD = 0.58	
